# N-Acetylgalactosamine Positive Perineuronal Nets in the Saccade-Related-Part of the Cerebellar Fastigial Nucleus Do Not Maintain Saccade Gain

**DOI:** 10.1371/journal.pone.0086154

**Published:** 2014-03-06

**Authors:** Adrienne Mueller, Adam Davis, Steven S. Carlson, Farrel R. Robinson

**Affiliations:** 1 Department of Biological Structure, University of Washington, Seattle, Washington, United States of America; 2 Department of Physiology and Biophysics, University of Washington, Seattle, Washington, United States of America; Tokai University, Japan

## Abstract

Perineuronal nets (PNNs) accumulate around neurons near the end of developmental critical periods. PNNs are structures of the extracellular matrix which surround synaptic contacts and contain chondroitin sulfate proteoglycans. Previous studies suggest that the chondroitin sulfate chains of PNNs inhibit synaptic plasticity and thereby help end critical periods. PNNs surround a high proportion of neurons in the cerebellar nuclei. These PNNs form during approximately the same time that movements achieve normal accuracy. It is possible that PNNs in the cerebellar nuclei inhibit plasticity to maintain the synaptic organization that produces those accurate movements.

We tested whether or not PNNs in a saccade-related part of the cerebellar nuclei maintain accurate saccade size by digesting a part of them in an adult monkey performing a task that changes saccade size (long term saccade adaptation). We use the enzyme Chondroitinase ABC to digest the glycosaminoglycan side chains of proteoglycans present in the majority of PNNs. We show that this manipulation does not result in faster, larger, or more persistent adaptation. Our result indicates that intact perineuronal nets around saccade-related neurons in the cerebellar nuclei are not important for maintaining long-term saccade gain.

## Introduction

Perineuronal nets (PNNs) are features of the CNS extracellular matrix that surround synaptic contacts on the soma and dendrites of neurons (reviewed by [1]–[3]). PNNs are composed of several molecules, including hyaluronan, link proteins and chondroitin sulfate proteoglycans (CSPGs) (reviewed by [2]–[4]). There are several different molecular species of CSPGs present in PNNs, the most common of which is aggrecan (reviewed by [2], [5]). CSPGs consist of a protein core and a variable number of chondroitin sulfate side chains (CS chains) (reviewed by [1], [6]. CS chains are composed of a polymerized disaccharide unit of uronic acid and sulfated N-acetylgalactosamine [7].

Previous reports show that the appearance of PNNs correlates with the end of developmental critical periods [8]–[10]. Critical periods are specific time periods early in life during which the brain is very plastic (reviewed by [11]). That is, during a critical period experience shapes synaptic organization. The formation of PNNs may contribute to ending the plasticity of critical periods by inhibiting plasticity (reviewed by [2], [3], [6], [12]). Several recent studies reestablish critical period-like high levels of plasticity by enzymatically digesting CS chains in perineuronal nets of adult rats [9], [13], [14] and mice [15].

PNNs surround a higher percentage of neurons in the cerebellar nuclei (97.6% in mice [16], 96% in rat [17], 93% in rhesus monkey, unpublished data) than in any other part of the brain (e.g. <10% in primate cerebral cortex, unpublished data; 5.6% in mouse barrel cortex [10]; 64–81% in human spinal cord [18]). The high prevalence of PNNs in the cerebellar nuclei raises the possibility that PNNs strongly inhibit plasticity in the cerebellar nuclei. If so, then enzymatically digesting CS chains in perineuronal nets in the cerebellar nuclei may reinstate critical-period-like plasticity as previously demonstrated in other parts of the brain [9], [14]. We would expect new experience to affect movements, because the cerebellar nuclei modulate the activity of premotor networks in the brain stem (reviewed by [19]). The activity of neurons in the cerebellar nuclei correlates with movements and damage to the nuclei impairs movements (reviewed by [19]).

Perineuronal nets form around neurons in the cerebellar nuclei during the first three months of development in monkeys [20]. A particular part of the cerebellar nuclei, the caudal part of the medial or fastigial nucleus, or CFN, strongly influences saccades [21]–[25]. During the period prior to the formation of PNNs, rapid eye movements, saccades, in young monkeys become progressively less variable and more accurate (Phillips et al, personal communication). It is plausible that during this same period the visual experience of inaccurate saccades shapes the synaptic organization in the CFN to make saccades less variable and more accurate. In this view, once the brain achieves the organization appropriate to producing accurate saccades, PNNs form around CFN neurons to stabilize the appropriate connections.

In the work described here we sought to implicate PNNs in the CFN in saccade plasticity. We knew that this system is amenable to pharmacological intervention, since CFN inactivation with the GABA-agonist muscimol abolishes saccade adaption [26]. To implicate PNNs, we digested CS chains in a monkey's CFN by injecting the enzyme ChABC into the CFN. ChABC depolymerizes the CS chains from the core proteins and hydrolyzes hyaluronan [27], but leaves the core proteins intact [28]–[30]. After we injected ChABC we provided the monkey with visual experience indicating that its saccades were consistently too small. We provided this experience several hours/day for consecutive days. This is a long-term saccade adaptation protocol, described previously [31], [32], which causes slow accumulative changes in saccade size over ∼20–30 days of experience. We compared the size and rate of the change in saccade size when the CS chains in the monkey's CFN were intact and after we digested them.

In a control experiment, we injected ChABC into regions of this monkey's cerebellar nuclei outside the fastigial nucleus and, after we sacrificed the animal, stained sections of the cerebellum for fully intact PNNs. We used this histology to estimate the size of the area within which our injections into the CFN digested CSPGs. These data confirmed that our injections into the CFN affected PNNs within the entire CFN. We made these control injections at different times before sacrificing this animal, allowing us to describe how quickly PNNs returned to normal after ChABC treatment.

## Methods

### Animal Preparation

We implanted one male rhesus macaque (Monkey S, age 9 years) with scleral search coils in both eyes using established methods [33], [34]. Briefly, we made a circular incision in the sclera just peripheral to the iris and placed a coil of thin Teflon coated wire into this opening. We led the ends of the coil's wires laterally and under the skin to the top of the skull, where we attached them to a small plug. We used dental acrylic to secure the plug to the animal's skull with small screws. In the same surgery we also implanted acrylic lugs on the monkey's skull allowing us to stabilize its head during eye movement recording.

In a second surgery we also implanted a recording chamber centered on the midline aimed directly ventrally 8 mm posterior to ear-bar zero. This put the center of the chamber directly dorsal to the midline between the left and right CFNs.

For both surgeries, we first sedated the animal with intramuscular injection of Ketamine HCl. We then induced anesthesia with intravenous Propofol, 2–5 mg/kg and after that intubated and anaesthetized to surgical plane with inhalation anesthetic (Isoflurane or Sevoflurane 1–2%). The animal also received intravenous Fentanyl Citrate (10–20 µg/kg) during surgery. Surgeries were performed in aseptic conditions. After surgery the monkey received intramuscular injections of Ketoprophen 5 mg/kg, and/or Buprenorphin, 0.015 mg/kg every 8 hours continued at the discretion of attending veterinarians.

All of our procedures were specifically approved by the University of Washington Animal Care Committee (Protocol 2340-01) and conformed to the recommendations from the Institute of Laboratory Animal Resources and the American Association for Accreditation of Laboratory Animal Care.

Monkey S was housed in a cage that allowed social contact with the animal in the neighboring cage. This contact was disallowed during periods shortly leading up to and while the animal was goggled. This was to ensure that Monkey S did not suffer mistreatment from the neighboring animal while he couldn't see. All animals on our protocol, including this one, are fed a daily regimen of protein biscuits in addition to the apple-sauce they receive as food reward for completing saccades tasks. They have unrestricted access to water. Animals receive weekly environmental enrichment in the form of toys, puzzles, and novel foods. Animals are monitored daily by research staff, animal technicians and/or veterinary staff. Any sign of discomfort or illness is brought to the attention of the veterinary staff and animals are treated appropriately to alleviate the condition.

### Long-Term Saccade Adaptation

We trained the animal to make saccades for apple-sauce reward. After the monkey became proficient at this task, it underwent two consecutive long-term adaptation and recovery experiments separated by two weeks. During the first experiment the PNNs in the monkey's CFNs were unaffected. At the beginning of the second experiment, we injected ChABC bilaterally into the CFN to digest CS chains of PNNs.

### Eliciting and Measuring Long-Term Adaptation

We measured the size of saccades using the eye coils implanted in the procedure described above. When the coils are inside the magnetic field of our booth the field induces a small current in the coil. The size of the current depends on the angle between the coil and the field. When the monkey rotates its eyes, that angle changes and the current induced in the coils changes. We recorded these currents via the plug on the top of the monkey's head and converted them into voltages proportional to the direction of the monkey's eye.

To elicit long-term saccade adaptation we used a technique modified from McLaughlin's [35] method for adapting (changing) saccade size. We trained the monkey to make saccades to track the horizontal movement of a small (0.3°) target spot. During each saccade, the target moved again farther from its starting point. Thus, at the end of each saccade the eye seemed to have fallen short of its target. When a monkey makes repeated saccades to targets that moved like this, the size of its saccades gradually increases [36]. When a monkey tracks such target movements for only a few hours, the elicited change in saccade size fades quickly when the monkey subsequently makes saccades to track normal targets, i.e., ones that do not move during saccades, or even when the monkey makes no visually guided saccades [37]. In contrast, if we repeat this adaptation procedure for multiple days, blindfolding the monkey with goggles between its daily adaptation sessions, then saccade size increases day to day [32]. The goggles prevent visually guided saccades outside the booth. In this condition the only saccades that the monkey makes are to targets that move during the saccade, so the monkey retains some of the change from previous training. Changes in saccade size elicited by this long-term procedure persist longer than do changes caused by a single day's adaptation [31], [32].

We used the long-term saccade adaptation procedure to adapt Monkey S to make 16° saccades when presented with targets 8° to the left or right of where its eye aimed. We measured saccade accuracy by calculating the gain of each saccade. Gain is saccade amplitude divided by initial target amplitude. Thus, for our experiment the gain of its saccades went from ∼1 to ∼2. We adapted Monkey S's saccades to both the left and the right but, for brevity, we show only the results of leftward adaptation experiments in this article. The results for rightward adaptation were very similar.

**Figure 1:**
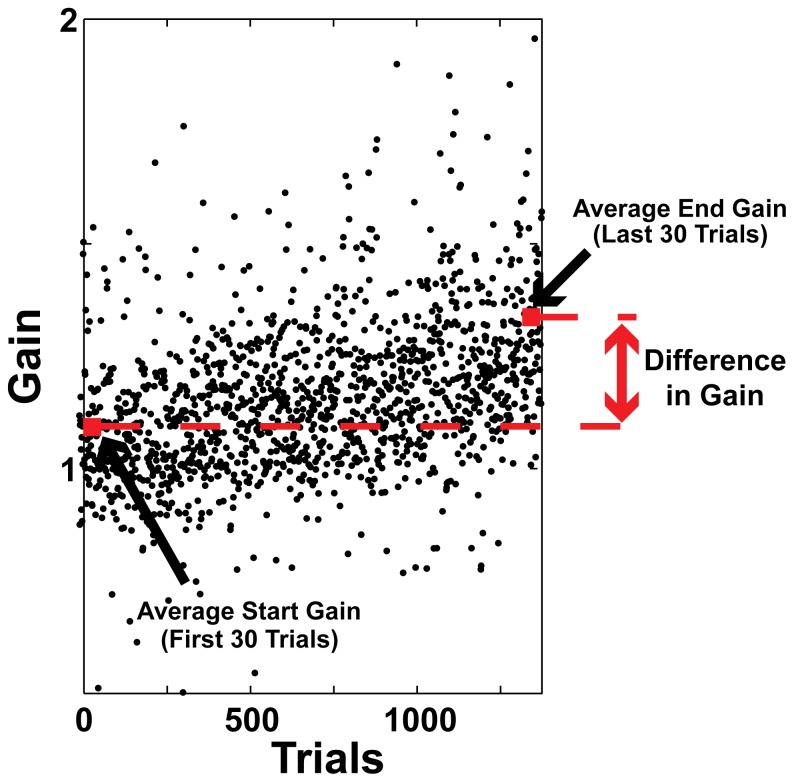
Difference in gain. The difference in gain is calculated by subtracting the average start gain of the first 30 trials from the average end gain of the last 30 trials.

We measured the progress of adaptation by calculating daily gain change. This is the average gain of the last 30 saccades in a daily adaptation session minus the average gain of the first 30 trials of that session. Figure 1 shows typical data from one daily adaptation session and how we calculated the daily gain difference. We can then fit an exponential curve of the formula Fit(g) = b ± A * e^(n/T)^ to the daily difference in gain values. The time constant, _T_, of this exponential curve is an estimate of the rate of gain change during adaptation or recovery from adaptation. The other variables represent the differences in gain (g), the bias (b) and the number of days the adaptation or recovery took (n).

Our long-term adaptation training of Monkey S continued until the average gains of the first 30 trials and the last 30 trials in a session were significantly the same in at least one direction. We use the TOST equivalence test to determine whether the average start gain and average end gain were significantly the same [38]. We then continued to present the animal with adapting target steps for a further two weeks (consolidation period). This allowed time for the perineuronal nets that we manipulated in the second experiment to re-form.

After these two weeks, we again presented normal 8° target movements (i.e., no target movement during saccades) to the animal during its daily adaptation sessions while still blindfolding it between sessions. This procedure, which we called Recovery, elicited a gradual decrease in saccade gain toward the normal value of 1. Recovery training continued for consecutive days until the averages of the first and last 30 saccades in a session were significantly the same in at least one direction.

To compare the saccade gains during our two conditions, i.e., when PNNs were intact and when we disrupted them by injecting ChABC, we performed the standard two-tailed students T-test and assumed statistical significance at p-values less than 0.05.

### Injection Procedure

We used epoxylite and polyamide-coated extracellular tungsten electrodes with impedances of ∼300 kΩ to record from neurons in Monkey S's cerebellar nuclei. We introduced the electrodes into the cerebellum through a guide cannula that ended above the cerebellar nuclei. We identified the CFN by the characteristic burst of action potentials that neurons there exhibit for every saccade. Once we located the CFN on both sides of the cerebellum we drove an injection pipette into this region bilaterally and injected between 1 and 1.6 µl of a solution of (60 U/ml) chondroitinase ABC (100330 from Seigaku Corporation) dissolved in 0.9% serological saline. We made bilateral injections on days 1, 4 and 7 of the second long-term adaptation experiment. The pipette consisted of an approximately 200 µm wide stainless steel hypodermic tube introduced into the brain through the same guide cannula that we used for the extracellular electrodes.

Before performing either electrode or pipette penetrations, we numbed the exposed area in the chamber by bathing it in 10 ml of 20 mg/ml Lidocaine for 10 minutes.

### Saccade Comparisons

In addition to measuring adaptation of saccade gain, we also compared the properties of saccades made both before and after we exposed CFN PNNs to the ChABC enzyme. We calculated the saccade velocity and acceleration: the first and second derivatives of the saccade position trace with respect to time. We measured the peak velocity, peak deceleration, and deceleration duration of each saccade in our sample.

To compare the values of these attributes when PNNs were fully intact and when ChABC was injected into the CFN, we performed two-tailed T-tests with Bonferroni correction and considered p-values less than 0.05 indicative of significant differences. We compared samples of saccades that were in the same direction, within 0.1° of a given amplitude and within 1° of given starting position. We also removed the saccades within the lowest 20% of the velocity range of a set that did not match typical velocity or acceleration profiles.

### Histology

To confirm that our ChABC injections digested CS chains in the primate cerebellar nuclei, we made injections into four sites in the cerebellar nuclei outside of the CFNs. At each of these extra-CFN sites we injected the same volume of ChABC solution with the same three-injection time schedule that we used for the CFN injections. These additional sites were in the interpositus and dentate nuclei. We injected at each of these additional sites starting at a different times (28, 21, 14, and 7 days) before we sacrificed the monkey. We made two electrolytic lesions at each injection site using negative current of 30A for 30 seconds, electrode negative. At each site, we made two marking lesions 1 mm below the injection site on the first day of injection and 1 mm above the site on the last day of injection.

We sacrificed the animal by delivering a lethal dose of Nembutal to the already sedated animal and then perfusing it through the heart with 4% paraformaldehyde followed by 10, 20, and 30% solutions of sucrose in phosphate buffer. We used a cryostat to cut 25 µm frozen sections of the cerebellar nuclei and divided our sections into 4 sets. Thus consecutive sections within one set were 100 µm apart.

To verify the location of our marking lesions we stained one set of sections for Nissl. We mounted sections on charged slides, dehydrated them with increasing concentrations of ethanol, stained them with toluidine blue at pH 4.1 for ∼45 seconds, and then rehydrated them. We then dried sections on the slides and cover-slipped them.

We stained another set for intact perineuronal nets with wisteria floribunda agglutinin (WFA) conjugated with fluorescein (1∶500, Vector Labs FL-1351) using standard immunohistochemical techniques [39]. WFA is a lectin that recognizes sugar moieties on CS chains of chondroitin sulphate proteoglycans, CSPGs are primary components of perineuronal nets. ChABC digestion removes the binding sites for WFA. We therefore use WFA as a marker of fully intact PNNs, and the lack of WFA staining to track where CS chains have been enzymatically removed from their core proteins. We also stained this set with avidin conjugated with Texas Red (1∶500, Millipore Corp MAB377) to mark cerebellar nuclear neurons. This is not a standard neuronal marker, but others have shown that it labels cerebellar nuclear neurons when other more traditional stains do not [40]. To evaluate PNNs in the vicinity of our ChABC injections we made fluorescent images with Zeiss Axioskop 2 confocal microscope using LSM 5 Pascal software.

**Figure 2:**
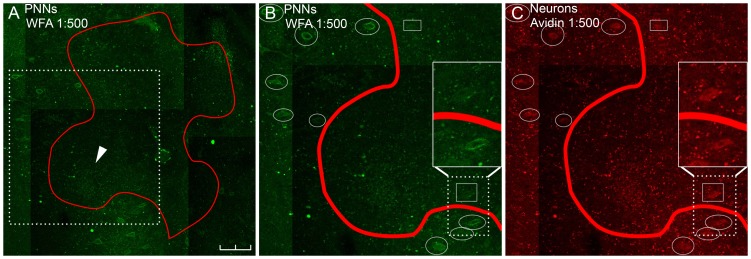
Identification of spread of ChABC. A: The extent of one lesion site is traced in red. PNNS are labeled with WFA (green). The white box indicates the region magnified in panels B and C. Scale bar = 200 µm. We frequently see nonspecific, punctate autofluorescence (arrowhead). This is a common phenomenon in adult primate brain tissue [40]. This can be extremely bright near the site of an electrolytic lesion. This is not indicative of PNN presence or absence because the stain does not form net-like circles around neurons. B: Magnification of square region in A, WFA-stain for PNNs. C: Magnification of square region in A, Avidin-stain for cerebellar nuclear neurons [41]. In both B and C, white circles surround neurons with WFA-staining and small white squares surround neurons without WFA-staining. Insets: magnified view of two neurons. The upper neuron does not have WFA-staining and is therefore is included in the injection site. The lower neuron does stain with WFA and is therefore excluded from the injection site.

We estimated the borders of the region within which ChABC digested CSPGs, by identifying the center of the injection sites relative to our marking lesions in the Nissl-stained set and then staining for both intact PNNs and for neurons as described above. We traced a border by working outward from the putative injection site until we found neurons surrounded by intact perineuronal nets (Fig. 2). In some areas we could not verify that neurons were present, but did not have fully intact PNNs, because of tissue damage caused by our injection (Fig. 2A, arrowhead). In these situations we extrapolated where to trace the border based on what we saw at nearby locations. Four individuals estimated the border location without knowing which injection they were examining or where anyone else put the border. We placed the border at the consensus of these estimates. Estimates from different individuals were similar.

## Results

**Figure 3:**
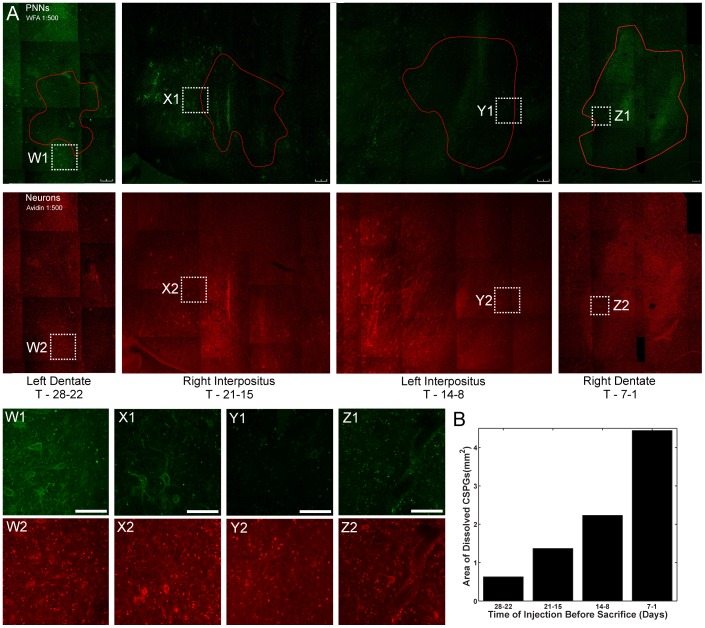
Degradation and reformation of PNNs in the cerebellar nuclei after exposure to ChABC. A: Degradation and reformation of PNN CSPGs in the cerebellar nuclei. Top panels show staining of four different areas of the cerebellar nuclei for CSPGs of perineuronal nets with WFA-Fluorescein. The proposed maximal extent of the spread of the enzyme is indicated in red. The time of the injections before sacrifice of the animal is noted below each column in days. Bottom panels shows the same areas stained with avidin to indicate locations of cerebellar nuclear neurons. Scale bars = 200 µm. Note that the right dentate is at a different scale to accommodate the entire injection. Small regions for which we lacked a photo are filled in with black. Additionally W, X, Y and Z label locations within each image for which we include higher resolution views below. Scale bars = 100 µm B: Estimate of spread of ChABC. Calculated area (in mm^2^) without WFA-staining from A for each injection site.

ChABC degrades CSPGs of perineuronal nets in macaque cerebellar nuclei Table 1 shows the size of the injections into the four sites outside the CFN. We found that our ChABC injections completely digested CS chains of CSPGs in perineuronal nets 7 days after our injection series started. PNNs were still affected at 14 days. By 21 days the CSPGs had started to reform and by 28 days they were present in areas very close to or within an injection site (Fig. 3A, leftmost panel). We conclude that PNNs in the CFN were not fully intact during a significant proportion of long-term adaptation and that they re-formed well before the end of adaptation.

**Table 1:** ChABC injection schedule, volumes and locations.

Location	Days before Sacrifice	Volume of 60 U/ml ChABC (µl)
Left Dentate	28	1.2
	25	1.2
	22	1.2
Right Interpositus	21	1.32
	18	1
	15	1.2
Left Interpositus	14	1.2
	11	1.2
	8	1.3
Right Dentate	7	1.2
	4	1.26
	1	1.2

We measured the distance from the presumed center of an injection site within which ChABC altered PNNs (Fig. 3B, Table 2). There was no evidence of CS chain presence as detectable by WFA in the right dentate one day after the end of a series of three injections. The enzyme altered PNNs over an area of approximately 4.4 mm^2^ and within 1 mm horizontal distance from the presumed center of the injection site (i.e. ∼2 mm between the medial and lateral borders of the injection site). Each CFN is <1 mm in diameter; therefore we can infer that we altered perineuronal nets throughout the majority of the CFN.

**Table 2:** Extent of ChABC spread.

Location	Area of Spread (mm^2^)	Maximum Horizontal Spread (mm)	Maximum Vertical Spread (mm)
Left Dentate (T – 28-22)	0.63	0.78	0.97
Right Interpositus (T – 21-15)	1.37	1.12	1.32
Left Interpositus (T – 14-8)	2.23	1.53	1.79
Right Dentate (T – 7-1)	4.44	2.14	3.02

### ChABC injection did not change long term adaptation of saccades

**Figure 4:**
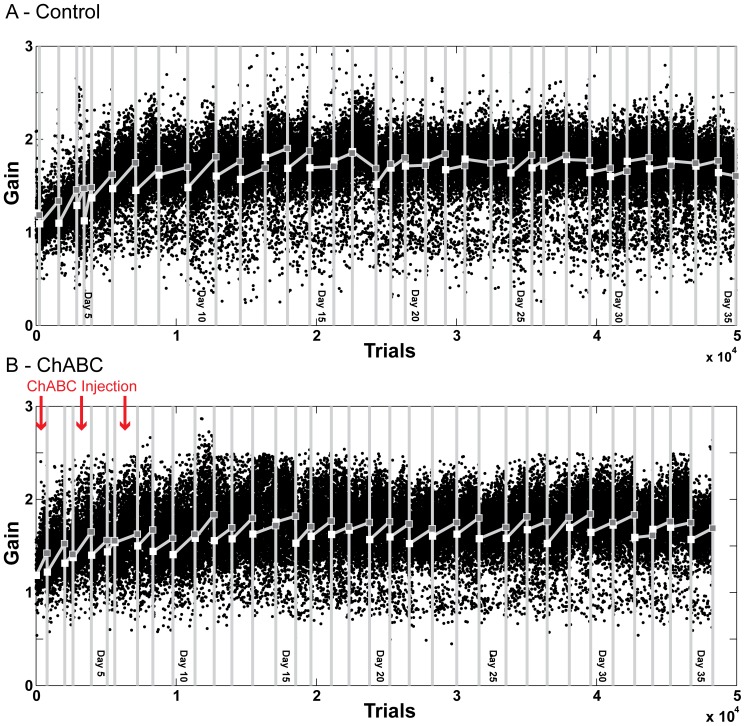
Long term saccade adaptation in Monkey S. A: Long term adaptation of saccade gain with PNNs fully intact. B: Long term adaptation of saccade gain with CFN ChABC-exposed PNNs. ChABC injections occurred on days 1, 4 and 7 of the experiment, red arrows. White squares indicate the average start gain (first 30 trials of a session) of each day's adaptation. Gray squares indicate the average end gain (last 30 trials of a session).

Figure 4 shows the gains of leftward saccades during 35 days of long-term adaptation both when PNNs were fully intact (4A) and when we injected ChABC bilaterally into the CFN on days 1, 3 and 7 of the long-term adaptation (4B). On day 1 we injected 1.6 µl into the left and 1.0 µl into the right CFN. On day 3 we injected 1.4 µl into the left and 1.6 µl into the right CFN. On day 7 we injected 1.4 µl into the left and 1.6 µl into the right CFN. There is no obvious difference in either the speed or magnitude of adaptation when PNNs were fully intact and after we injected ChABC into the CFN.

**Figure 5:**
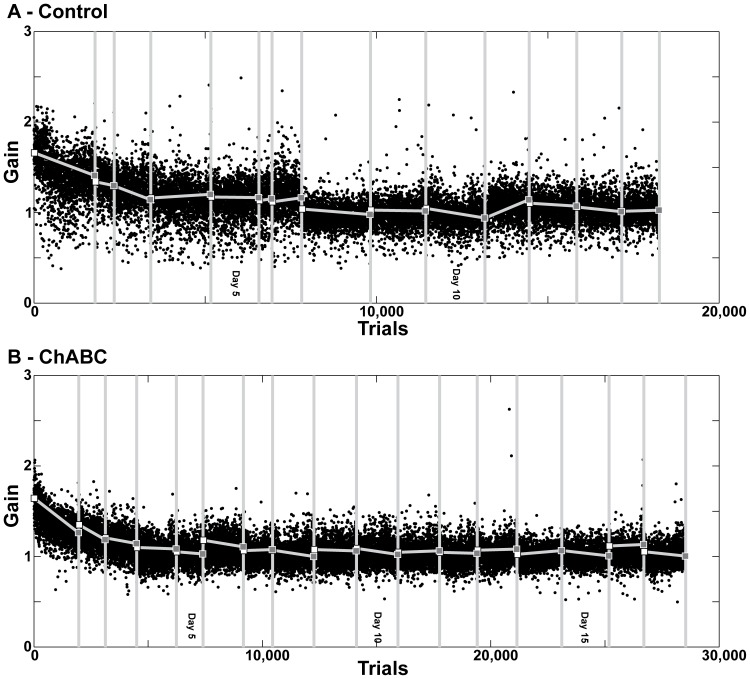
Recovery of long term adaptation. A: Monkey S' gain throughout recovery from long term adaptation with PNNs intact. B: Monkey S's gain throughout recovery from long term adaptation with ChABC treatment. White squares indicate the average start gain (first 30 trials of a session), gray squares indicate the average end gain (last 30 trials of a session).

Figure 5A shows saccades gains during recovery when CFN PNNs were intact and 5B shows gains during recovery after we injected ChABC. Like adaptation, recovery progressed at approximately the same rate in both conditions. This shows that an acquired saccade gain near 2 is not more persistent than normal after we digested CSPGs in the CFN and then let them re-form.

Before this experiment we expected that digesting the CS chains of CSPGs in the CFN during long term adaptation would result in either a larger change in gain, a faster rate of adaptation, or a more persistent change in gain. The size of adaptation was very similar whether PNNs were fully intact or not (average adapted gain of 1.64±0.25 when PNN were intact and 1.62±0.22 when we digested CS chains). These values are not significantly different (p = 0.35). A larger change in gain would manifest as the animal achieving a gain closer to the target gain of 2.0.

**Figure 6:**
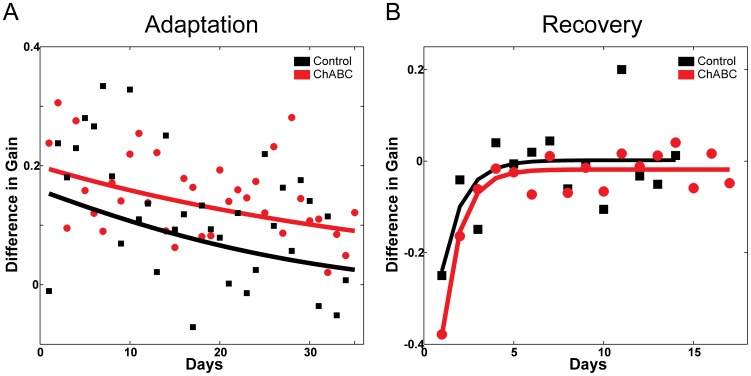
Rate of adaptation in control (PNNs intact) and ChABC conditions. A: Difference in gain for each day of long term adaptation and consolidation in both control (PNNs intact, black) and ChABC (PNNs altered, red) conditions. An exponential was fit to both sets of data. B: Difference in gain for each day of recovery in both control (PNNs intact, black) and ChABC (PNNs altered, red) conditions.

To measure the effect of injecting ChABC into the CFN on the rate and persistence of long-term saccade adaptation, we compared daily gain change during adaptation in both conditions (Fig. 6). We fit an exponential curve to these values for each condition. The time constant of the fit exponential represents the rate of gain change. As Figure 6A shows, the rate of change in both conditions varies considerably day to day and was similar in both conditions (time constants of −29.07 and −44.44 days respectively). The animal did not adapt more quickly (have a smaller time constant) when PNNs in the CFN were exposed to ChABC.

We anticipated that as PNNs recovered to their normal state around CFN neurons during adaptation, synapses appropriate to the new gain level are being maintained. If this were the case, then we should see a slower rate of recovery (return toward gain of 1.0) after we injected ChABC. Figure 6B shows that adapted gain recovered at very similar rates when PNNs in the CFN were intact or after we treated them with ChABC and allowed them to reform (time constants of 1.15 and 1.02 days respectively).

### ChABC injection did not impair saccades

PNNs surround a large proportion of neurons in the cerebellar nuclei. Without more information about PNN function, it is possible that injecting ChABC in the CFN could impair CFN function. To assess how well the CFN functioned after ChABC exposure we examined saccades at three times during each experiment, 1) before adaptation, 2) during days 10–14 of long-term adaptation, and 3) during days 10–12 of recovery. We limited our examination to saccades within a very narrow size range as described in Methods.

**Figure 7:**
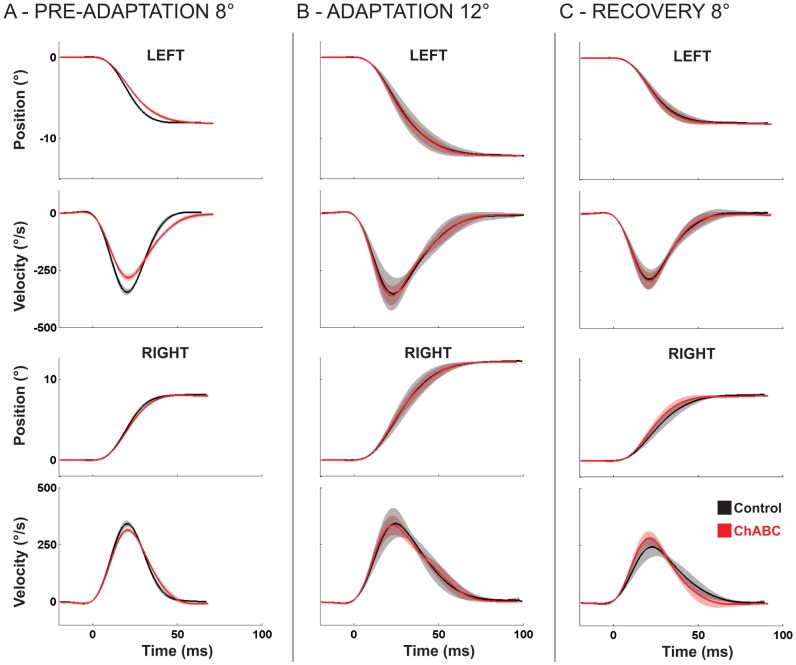
Comparison of control (PNNs intact) and ChABC saccades. A: Pre-Adaptation comparison of position and velocity profiles of leftward (top) and rightward (bottom) 8° saccades for control (black) and ChABC injection (red) experiments. Traces are averages (line) of several saccades with shading for standard deviation. B: Same as A, for 12° saccades pooled across days 10–14 of long term adaptation. C: Same as A, for 8° pooled across days 10–12 of recovery.

We found that, as Figure 7 and Tables 3 and 4 show, both leftward and rightward 8°saccades exhibited significantly lower peak velocities before the long-term adaptation in which we injected ChABC than before the adaptation when PNNs were intact. Saccades before ChABC injection experiment also had significantly lower peak decelerations and longer deceleration durations. These changes are characteristic of impaired CFN function ([42], [43]). Given that we drove electrode tracts through the vicinity of the CFN before the ChABC long-term adaptation, we believe that the saccade abnormalities we observed before the ChABC adaptation were a result of mild damage to the CFN caused by our electrode penetrations.

**Table 3:** Comparison of control and ChABC saccade values for leftward eye movements.

		PRE-ADAPTATION			ADAPTATION			RECOVERY		
		Peak Vel. (°/s)	Peak Dec. (°/s/s)	Dec. Duration (ms)	Peak Vel. (°/s)	Peak Dec. (°/s/s)	Dec. Duration (ms)	Peak Vel. (°/s)	Peak Dec. (°/s/s)	Dec. Duration (ms)
Control	Mean	−345.13	18830.77	24.71	−361.17	12841.84	50.00	−287.76	12789.19	33.27
	SD	15.10	1878.56	1.25	60.23	5286.12	71.31	42.15	3913.89	7.53
	N	7	7	7	106	106	106	114	114	114
ChABC	Mean	−282.93	11873.75	36.17	−361.27	12165.39	45.73	−294.26	13292.35	31.99
	SD	14.50	1411.85	6.74	41.22	3042.96	6.59	35.08	3537.12	7.56
	N	6	6	6	67	67	67	195	195	195
T-Test *B		* 7.30E-05	* 6.81E-05	* 0.05	5.94	1.72	3.25	1.00	1.21	1.26

Abbreviations: Peak Vel. = Peak Velocity, Peak Dec. = Peak Deceleration, Dec. Duration = Deceleration Duration. T-Test.

*B = Bonferroni corrected T-Test.

Values can be greater than 1, but p-values<0.05 are considered significant and demarcated with a *.

**Table 4:** Comparison of control and ChABC saccade values for rightward eye movements.

		PRE-ADAPTATION			ADAPTATION			RECOVERY		
		Peak Vel. (°/s)	Peak Dec. (°/s/s)	Dec. Duration (ms)	Peak Vel. (°/s)	Peak Dec. (°/s/s)	Dec. Duration (ms)	Peak Vel. (°/s)	Peak Dec. (°/s/s)	Dec. Duration (ms)
Control	Mean	342.05	−18829.22	25.92	353.95	−13310.55	44.31	251.61	−9717.01	38.15
	SD	14.69	1740.71	1.68	55.15	4014.68	5.03	35.44	3069.82	4.07
	N	12	12	12	64	64	64	54	54	54
ChABC	Mean	291.97	−13958.45	25.64	336.65	−10414.35	44.81	280.84	−11783.73	33.26
	SD	84.50	4206.47	7.72	40.99	2863.70	5.83	32.36	2929.04	7.29
	N	14	14	14	74	74	74	172	172	172
T-Test *B		* 1.55E-04	* 2.99E-05	0.29	0.25	* 2.85E-05	3.54	* 3.83E-06	* 2.14E-04	* 2.33E-08

Abbreviations: Peak Vel. = Peak Velocity, Peak Dec. = Peak Deceleration, Dec. Duration = Deceleration Duration. T-Test.

*B = Bonferroni corrected T-Test.

Values can be greater than 1, but p-values<0.05 are considered significant and demarcated with a *.

To evaluate saccades during adaptation we examined saccades that the monkey made during days 10 to 14 of adaptations both with and without ChABC exposure, and on days 10 to 12 of both recoveries. We found that saccades made after we injected ChABC did not exhibit significantly lower peak velocities, lower deceleration rates, or longer decelerations than compared to control saccades, (Fig. 7B, Tables 3, 4). Likewise, saccades during recovery after we injected ChABC were not significantly different from control saccades during control recovery. (Fig. 7C, Tables 3,4). Thus the only saccades that showed signs of CFN dysfunction were before we injected ChABC into the CFN. We conclude that ChABC exposure did not impair PNN function as reflected in saccades.

## Discussion

This study shows that digesting the CS chains of CSPGs in CFN perineuronal nets does not affect the rate, size, or persistence of long term saccade adaptation. Further, digestion does not impair CFN function as reflected in several saccade attributes sensitive to CFN dysfunction [43]. Our control injections outside the CFN show that we affected perineuronal nets in the CFN, and that the nets had sufficient time to recover during the two week consolidation period of the long term adaptation. Therefore, we conclude that the extensive GAGs on perineuronal net CSPGs around CFN neurons are not important for setting saccade gain.

Perineuronal nets surround more neurons in the cerebellar nuclei than anywhere else in the brain. If ChABC sensitive CSPGs in the CFN are not inhibiting plasticity of saccade size, then what could they be doing? Others have suggested that the role of perineuronal nets is not purely to inhibit plasticity. It is possible that they surround highly active cells (such as cerebellar nuclear neurons, which fire at a baseline rate of 50–100 Hz) to provide a cation sink [44], [45], reviewed by [46]. In this view the presence CS chains in PNNs is not tied directly to modifying the movement-related output of nuclear neurons, but instead aids their cellular physiology.

Still, our result clearly contrasts with other studies in which digesting CS chains in perineuronal nets strongly increased plasticity and changed performance. Pizzorusso et al [9], [13] showed that degradation of CS chains in V1 of an adult rat restores critical period levels of plasticity. This effect was present even though only a small fraction of neurons in the visual cortex was surrounded by WFA-positive perineuronal nets (unpublished data) and thus likely to be affected. Gogolla et al.'s study [14] showed that digesting CS chains in the amygdala allows extinction of the conditioned fear response in adult rats. This extinction is normally only possible in young rats. Like V1, the amygdala contains only a small proportion (∼2%) of neurons surrounded by WFA-positive PNNs (unpublished data). One way to reconcile our data with these previous studies is to propose that, in the CFN, fully intact perineuronal nets are necessary, but not sufficient to end plasticity. Perhaps an additional factor contributes to inhibition of synaptic plasticity. Thus, unlike visual cortex, other components need to be reversed in the CFN to restore plasticity. The degradation of the PNN CS chains alone is not enough.

Another possibility is that PNNs around CFN neurons mediate plasticity of some features of saccades but do not directly contribute to the change in gain during long term adaptation. We think this possibility is less likely because previous work shows that 1) CFN cell activity modulates with at least short-term saccade adaptation [47] and 2) CFN inactivation with the GABA-agonist muscimol abolishes short-term saccade adaption [26].

Alternatively, CSPGs in the CFN might be reduced during long-term adaptation in mature animals under normal conditions. In this case, our application of ChABC would not necessarily influence the rate, magnitude or permanence of long-term adapted saccades any further. Recent studies show that PNNs change during plasticity. Sale et al (2007) [48] found a reduced density of PNNs in the visual cortex of amblyopic adult rats that received environmental enrichment which restored their visual acuity. Foscarin et al (2011) [16] showed a similar effect in the cerebellar nuclei of mice: environmental enrichment reduced PNNs by approximately 17%. Further, Deák et al (2012) [49] showed reduced PNN presence in the vestibular nuclei of rats that underwent unilateral labyrinthectomy. However, if partially reducing PNNs, as these other studies have shown, allows plasticity in the CFN which facilitates adaptation, one would imagine that reducing them still further would allow even more plasticity and therefore change the profile of adaptation. Further studies are necessary to explore the extent to which PNNs around CFN neurons change during saccade adaptation, and whether application of ChABC allows additional plasticity in surfeit to physiological levels. Additionally, more research is needed to establish whether the cellular mechanisms that increase plasticity physiologically (as in [16], [48], [49]) are the same as those that occur after neurons are exposed to ChABC.

A final possibility that could account for our results is that our manipulation did not affect the right component of the PNN to cause changes in long term saccade adaptation. Indeed, a recent paper by Carulli et al [50] suggests that it is a decrease in the link protein Ctrl-1, and not necessarily CSPGs at all, that is permissive for plasticity. Also, although ChABC cleaves the GAG chains of N-acetylgalactosime positive CSPGS, it leaves the core proteins intact [51]. If a GAG-independent affect of the CSPG core protein is the crucial component for plasticity, we will not have altered it with our manipulation. Further studies are necessary to determine whether digestion of CSPGs, or PNNs as a whole, are necessary for increased plasticity in the cerebellar nuclei.

Some of our data is consistent with the possibility that degrading CS chains of CFN PNNs allows repair of saccade speed and deceleration. In the CFN prepared for ChABC injection (but before injection and adaptation) saccades were slower than in the normal CFN. This mild impairment of saccades was likely due to a small amount of CFN damage caused by our electrode penetrations. After ChABC exposure, saccade velocity profiles returned to normal. They were indistinguishable from control saccades. The improvement of saccades from mildly impaired before ChABC injection to normal after injection suggests that digesting the PNN CS chains allowed synaptic reorganization repairing electrode penetration damage.

If electrode damage had been extensive enough to significantly impair CFN function, then our ChABC injections would not have had a substrate on which to act. We do not think that this happened. If it had, then saccades would have been more impaired than they were and Monkey S would not have been able to adapt at all.

There are at least two alternative explanations for improvement in saccades during and after ChABC injection. First, the improvements in saccade peak velocity and deceleration duration after injection might result from other compensatory mechanisms that operate with or without digesting CFN PNN CS chains. Second, it may be that, Monkey S's saccades are so variable these results are an artifact of the small sample size of pre-adaptation saccades. Currently we can conclude only that our results do not eliminate the possibility that digesting CS chains of PNNs in the CFN allowed improvement of slightly impaired saccades.

It is interesting to note that during development, CSPGs accrue within a period of about 90 days while in an adult monkey they re-assemble within three weeks. This means that production of PNN components is not restricted to a particular time period during development. This is consistent with previous studies that describe the recovery of perineuronal net CSPGs in other areas of the brain in other species [27], [52], [53].

## Conclusion

In summary, we show that digesting CS chains of perineuronal nets in the saccade-related part of the cerebellar nuclei, the CFN, does not significantly change the rate, size, or persistence of long-term saccade adaptation. Altering PNNs in the CFN or in other parts of the cerebellar nuclei had no noticeable effect on behavior. Apparently, these neurons function at least approximately normally for up to 21 days without fully intact PNNs. CSPGs reform within 21 days of being degraded in the adult primate brain. We have yet to understand the role of the dense PNNs in the cerebellar nuclei.
